# Snus user identity and addiction. A Swedish focus group study on adolescents

**DOI:** 10.1186/1471-2458-12-975

**Published:** 2012-11-13

**Authors:** Ingrid Edvardsson, Margareta Troein, Göran Ejlertsson, Lena Lendahls

**Affiliations:** 1 Department of Clinical Sciences, Malmö, Family medicine, Lund University, Jan Waldenströms gata 35, Malmö, SE-205 02, Sweden; 2 School of Health and Society, Kristianstad University, Kristianstad, SE-291 88, Sweden; 3 Unit for Research and Development, Kronoberg County Council, Box 1223, Växjö, SE-351 12, Sweden

**Keywords:** Adolescents, Addiction, Content analysis, Focus group, Snus user, Identity

## Abstract

**Background:**

The teenage years are the years when adolescents seek their identity, and part of this involves experimenting with tobacco. The use of tobacco as such, and norms among their friends, is more important to the adolescents than the norms of parents when it comes to using tobacco or not. The aim was to explore the significance of using snus for adolescents, and attitudes to snus, as well as the reasons why they began using snus and what maintained and facilitated the use of snus.

**Methods:**

Adolescents who use snus were interviewed in focus groups. The material was analysed using content analysis.

**Results:**

Four groups of boys and one group of girls were interviewed, a total of 27 students from the upper secondary vocational program. Three themes related to the students’ opinions on and experiences of using snus were found: *Circumstances pertaining to snus debut* indicate what makes them start using snus. *Upholding,* which focuses on the problem of becoming addicted and development of identity, and a*pproach*, where the adolescents reflect on their snus habits in relation to those around them. A number of factors were described as relevant to behaviour and norm building for the development into becoming a snus user. Attitudes and actions from adults and friends as well as – for the boys – development of an identity as a man and a craftsman influenced behaviour.

**Conclusions:**

The results showed that development of identity was of major importance when adolescents start using snus. The adolescents were initially unable to interpret the early symptoms of abstinence problems, but subsequently became well aware of being addicted. Once they were stuck in addiction and in the creation of an image and identity, it was difficult to stop using snus. These factors are important when considering interventions of normative changes and tobacco prevention in schools as well as among parents.

## Background

Adolescence has been identified as the period in life when experimentation with tobacco increases dramatically. Swedish studies indicated that the use of snus is introduced later than smoking among adolescents [1,2]. In grade 2 in upper secondary school (age 17), 24 per cent of the boys and seven per cent of the girls used snus in Sweden 2011, and the trend is slowly decreasing [3].

Smokeless tobacco use occurs in a number of countries around the world, and smokeless tobacco comes in a variety of ways. The products contain unhealthy substances at different levels, and nicotine, that get the user hooked on the addiction [4]. Snus (the traditional Swedish type of oral moist snuff) is forbidden for export outside Sweden. The use of snus increases the risk of reversible and irreversible oral lesions, ventricle, and oesophagus [5-8], and it also increases the risk of dying from a heart attack or stroke [9,10]. Using snus during pregnancy increases the risk of premature delivery and pre-eclampsia [11].

Social identity and belonging to a group are important during the adolescent years, a time when it is common among teenagers to experiment with tobacco. This could be part of the adolescent seeking his/her identity. Friends have an important role in a young person’s life, and the group norms, attitudes and behaviour of the friends have a stronger impact on the adolescent than those of his/her parents [12]. According to Tajfel's theory, a social identity is created in three steps. The first is categorization, the grouping of yourself in a hierarchy based on how you feel other people see you, secondly identification when you are compared with others, and - finally - by social comparison when you identify yourself with other people in the same group, such as those who have the same profession or nationality, and where you perceive your own group in a more positive way than other groups [13].

Why adolescents start using tobacco is determined by multiple factors. According to Ajzen’s Theory of Planned Behaviour (TPB), behavioural intention is the most important predictor of behaviour. Behaviour intention is predicted by attitudes towards the behaviour, subjective norms, and perceived behaviour control. Each of these three variables reflects a set of underlying accessible beliefs, which include behaviour beliefs, normative beliefs, and control beliefs for perceived behaviour control [14]. More recent research on smoking has used TPB as a rule of thumb for understanding the motivation behind whether you start smoking or not. TPB demonstrated that the intentions to smoke were normally directed by attitudes and perceived behaviour control [15]. Another factor that complicates the picture of the onset of snus use is the development of nicotine addiction. Studies indicate that occasional smoking during adolescence can cause a rapid development of nicotine addiction, even before smoking becomes a daily habit [16]. A Swedish study showed that adolescents who are exclusively using snus had a two to fivefold higher risk of becoming addicted to nicotine compared to those who were only smokers [17].

As far as we know, there are no previous studies on adolescents’ experiences of snus usage.

The aim was to explore the significance of using snus for adolescents, and attitudes to snus, as well as the reasons why they began using snus and what maintained and facilitated the use of snus.

## Method

### Study population

The study was performed in the county of Kronoberg in southern Sweden, with 185 000 inhabitants in eight municipalities. There are 19 upper secondary schools in the county, both private and municipal ones. A majority of the students (66 percent) went to schools in the largest municipality with around 83 000 inhabitants.

### Study design

Focus group interviews are defined as a scientific method where data is collected through group interaction on a topic decided by the scientist [18]. This method was selected for studying the contents, i.e. the views, attitudes, opinions, and arguments the participants expressed in a group. Focus group interviews will also give insight into the ideas and concepts used in a cultural context [19]. The method gives a variety of opinions as well as close contact with the snus-using adolescents for increased knowledge without the purpose of reaching consensus or influencing them in any direction.

The participants were recruited through the school nurse who had a relatively good knowledge of the students' tobacco habits, as she had regular individual health discussions with them on general life habits. The school nurse received oral information by phone from the first author (IE) and instructions in writing on the selection. Inclusion criteria were that the participants used snus on a regular basis but did not smoke. They should not have a chronic illness such as asthma or diabetes, as health reasons may have influenced their choice to use snus instead of smoking. Adolescents using snus were invited into the study and received information in writing on the purpose of the group interview, on the procedure, and that all the material would be treated as strictly confidential. The adolescents gave written, informed consent to their participation. In total, 27 adolescents participated, aged 17–19, divided into five groups with four to six students in each group. Four groups of boys, and one group of girls from three schools were included in the study. The groups were based on the respective schools and the participants knew each other to some extent. They were all recruited from vocational upper secondary programs, such as building, farming, vehicle and animal care. As seen in a local questionnaire study, most snus users attended vocational programs [20].

IE acted as moderator of the focus group discussions during the interviews. The role of the moderator was to be prepared to guide the discussions if the group deviated from the subject, ask clarifying questions when necessary, and ensure that all the participants got a chance to voice their views. An assessor (LL) listened, observed, and took notes. Both were unknown to the participants. A guide with questions was developed with five different topics of interest: how they started using snus, circumstances that enabled the onset, students’ views on prevention, attitudes to snus use and speculations about the future. Each topic had open questions and they were constructed based on earlier questionnaires used in upper secondary school [20]. The interviews were conducted during school hours in a room at the students’ schools and lasted about 40–60 minutes each.

### Analysis

Qualitative content analysis was carried out with an inductive approach according to the Graneheim and Lundman theory [21]. This meant that meaningful units were identified, condensed and coded into categories. These categories were grouped according to content to form themes. This method was selected in order to create a clear and manageable picture of the participants’ experiences of being a snus user. The interviews were recorded on tape and transcribed verbatim by a secretary. IE listened to the recorded interviews and took notes to describe the feelings and the atmosphere of the interview situations. The material was read through a number of times to catch the overall feeling by IE and LL. To be true to the context, the meaningful sentences were condensed to a description close to the text, the manifested content, as well as an interpretation of the underlying meaning, the latent content. To enable processing of the text, the analysis was done in different steps. Firstly, meaningful units from all interviews were identified, and these were then condensed and coded and finally put together into different categories. IE and LL conducted the interviews and analysed the text, independent of each other, and then the different steps of the analysis were discussed until consensus was reached. An example of the analysis process is shown in Table 1. To clarify the results, three themes were crystallized to a more abstract level on the basis of the categories. Representative quotations are presented in italics and the group origin is presented in brackets. Group 1–4 consisted of boys and group 5 of girls only.

**Table 1:** Example of the analysis process

**Meaningful unit**	**Condensation**	**Code**	**Category**
We use snus in the vehicle program. Truck drivers, vehicle mechanics, and construction workers	The trade you belong to is associated with snus use.	Snus is used in certain professions	Identity

### Ethics

Before the students were approached, the principals of the schools sanctioned the study at their schools. The participating adolescents were informed in advance, and at the time of interview, about confidentiality and that participation in the study was voluntary. Before the interviews started, the participants gave their written informed consent for participation. If any of the respondents needed support after the interview to give up their tobacco use, the school nurses would assist them.

The study was conducted in agreement with Swedish Laws on Research Ethics and was approved by the Regional Ethics Committee at Linköping University (approval number 175–09).

## Results

The analysis of the text content resulted in eight categories. Three themes were developed from these (Table 2). These themes took up areas about the circumstances influencing the adolescents when they started using snus and the factors making them continue to use snus, and what the snus means to them, their approach to their roles as snus users in relation to others and how they saw themselves in the future. As differences in opinions and views between boys and girls have been noted, they have been reported in separate groups. The related categories are indicated in **bold text** and illustrated by quotes for each theme to show the foundation for our interpretation of the focus group discussion data.

**Table 2:** The eight categories and three themes in the analysis

**Categories**	**Theme**
Influence from significant others	Circumstances
Availability	pertaining to
Parent reactions	snus debut
Caught in addiction	Upholding
Identity	
Perceived advantages	
Influence others	Approach
Ambivalence	

### Circumstances pertaining to snus debut

The most important circumstance pertaining to snus debut was the **influence from significant others**. All participants had been influenced to start using snus by friends who treated them to snus. It was rarely a conscious choice but something that “just happened”. The analysis showed that regardless of whether they were aware of it or not, there was a group of snus-using friends in the background who influenced them. Many adolescents stressed the importance of being part of the group, which resulted in trying snus, and the difficulty to withstand peer pressure. Being part of the snus user group gave a feeling of belonging and identity. Snus-using family members were also role models, and together with these, they got a feeling of belonging when they used snus.

“I was with friends who were using snus, and then they gave me some and I bought a box myself and then I had started”."

"Interview 1"

Many of the adolescents described how they had practised in different ways in order to be able to use snus. By gradually getting used to the snus, they had “practiced away” physical symptoms such as nausea and dizziness. Overcoming initial feelings of disgust when they had gotten used to it gave room for other strong feelings of it being super and cool. They used snus to impress, be tough and cool, to defy something that was “forbidden” and because they appeared more grown-up. When they had tried it a few times, they continued without reflecting on the fact that it had become a habit.

“Then you felt so bloody sick, but still, the next time you still wanted it and then you felt just as sick again… but then it’s like you get going on it”

"Interview 1"

The **availability** of snus enabled them to become snus users. The adolescents never felt it was difficult to get access to snus and described how family members, such as the father and brothers, would offer them snus. After telling their parents they were using snus, some parents even bought them snus if they were under 18. But most common was that friends bought it for them, as they did not want to get their parents involved. Sometimes, they could even buy snus themselves, as there were always sales points where IDs were not checked. It quickly spread among friends where these sales points could be found.

“You talk to those who are over 18 and then it’s not a problem; it’s fixed. That’s why you have older friends who can buy it.”

"Interview 4"

“He did not check my ID and he did know I was younger… so he told me to put it in my pocket before I went away.”

"Interview 3"

The **parents’ reactions** were feeble, and not as strong as what the adolescents had expected. As they thought the reactions would be stronger, it took time before they had the courage to tell the parents that they were using snus, and they hid the snus boxes and tried in various ways to conceal their snus use. Few parents reacted strongly, others were resigned, and the adolescents thought they did not bother. Their failure to react made it easier for the students to become snus users. Some had parents who encouraged them to use snus instead of smoking, as snus was seen as a more healthy alternative. Some of the girls had not told anyone that they had started using snus instead of smoking, as the parents had previously reacted very strongly to their smoking and they wanted to avoid a new discussion about their snus habits. At the same time, some felt that the parents would not be able to do anything about them using snus anyway. It was their own choice.

“I felt more motivated to quit before my parents found out, because I thought there would be a hell of an uproar at home, but when I noticed that they did not care, it felt like… to hell, it doesn’t matter.”

"Interview 2"

### Upholding

Fairly quickly, the students, especially the boys, felt that they had been **caught in addiction** to nicotine. The physical addiction appeared as abstinence problems and they experienced various symptoms, which were not easily understood as they did not know what abstinence meant. Some wished they had quit before they got stuck with an addiction, some had experienced that it was difficult to quit, while others thought they could quit whenever they liked. The habit of always having something under the lip made them put in snus even if they did not feel a craving. The adolescents also expressed a social addiction, feeling that they felt they belonged to a group and the feeling of belonging from using snus together. The fellowship feeling could also make it harder to stop using snus, and most of the respondents felt that a snus-free environment was a prerequisite for being able to quit. It was hard to resist classmates using snus, and thus also difficult to stop using snus as long as you went to school.

“There is nothing positive about using snus, you know. Really! You learn to like it as times goes on, sort of, and then you feel that you need another one. No… there is nothing positive whatsoever, really, but it’s the thing you kind of do and then it get you addicted to it.”

"Interview 3"

“That’s nearly all it’s about when you’re young, and it should be as cool as possible and when you think it’s super-cool and then as you get older you realize you’re stuck on it, so it’s not so cool anymore.”

"Interview 3"

The girls expressed a mental addiction where snus was a good way to reduce their bad temper or irritation. If they were sad or angry, they used more snus, but when they were happy they did not need as much. Both boys and girls reported that they used less snus if they were busy with something, such as fixing a car or tending to a horse. On the other hand, they used more snus if they were bored and had nothing to do.

“Yes… but I don’t use snus that much, I don’t take a lot of it, but when I feel that I am getting into a bad temper, I take some snus and then I can manage for another good while, and sometimes I don’t need any snus because I feel happy.”

"Interview 5"

To be using snus is considered an **identity**, something one should be for the rest of the life. Most of the boys saw themselves as snus users in the future, but they could still consider quitting if they were going to be parents. To use snus was considered something genuinely Swedish, something in our culture, and the general opinion was that no immigrants used snus. The boys felt they were very masculine, and there were also those who claimed that their girlfriends thought it was sexy with snus and that it suited them to be snus users. The snus box was seen as a masculine attribute, which created a feeling of belonging. Using snus was also something associated with the professions they had chosen, for example farmers, carpenters, and car mechanics. In general, the boys reacted to the fact that the girls were using snus, as they felt it was a manly behaviour. Furthermore, it did not appear very attractive. Smoking was considered silly and something girls did to be cool. The adolescents felt there was a clear difference between using snus and smoking, but they found it hard to express what was different. Snus users were seen as better by both sexes, while smokers were less valued and sometimes despised. The girls said there was a clear difference where “tomboys” used snus and “bimbos” were smokers. The girls who used snus wanted to stick out from the crowd and be a bit different.

“Well, it’s just… ah… it is manly!”

"Interview 2"

“Well, it’s just like that, that farmers should use snus.”

"Interview 4"

“Well, bimbo… these little girls… mammies’ girls, you see them… they smoke… they would probably not ever consider using snus, but I guess they are more into smoking…”

"Interview 5"

The adolescents felt using snus had a number of **perceived advantages**. In school, there are lots of rules around smoking, but not around using snus. Using snus undisturbed was possible during class, without bothering anyone, but as a smoker you were referred to a certain place, and having to smoke outside in rain and cold weather was not an attractive alternative. They also saw the advantage that you did not smell, which the smokers did. Using snus was not considered a risk – health risks were regarded as uncommon and not well known. When they had used snus for a while and become used to it, they experienced a number of positive effects, such as that it was tasty and relaxing, and it gave a feeling of performing better and being able to concentrate.

“It gives an extra energy kick.”

"Interview 4"

“In school, you are not allowed to smoke– but it is okay to use snus, and you just throw it in the bin.”

"Interview 4"

“People around you are not harmed. Snus doesn’t smell and you don’t see it under the lip.”

"Interview 3"

### Approach

Even though the adolescents themselves had been **influenced by others** to start using snus, they did not feel that they influenced younger people to start. When the boys were occasional snus users, they were treated to it by snus using friends and never had to ask for it. As more established snus users, they never offered someone else snus, but treated their friends when they asked. Even if the adolescents felt grown-up when they started using snus, they did not want to give snus to younger children (around 12) as they did not want to contribute to their addiction. They were supposed to be older, around 15, and snus users already to be treated to it. The adolescents thought that it was up to the younger children to make their own choice about using snus. Friends who smoked were encouraged to switch over to snus as it had more advantages. One opinion they wanted to convey to younger children was that if they were to do something, they should use snus as it was considered less harmful. At the same time, they wanted to dissuade them from starting, and most of them felt it was stupid to start something that made you addicted and cost money. This was something they had not fully comprehended when they started.

“We normally treat those who ask… it’s only because you want to be kind, and they will have to face the consequences if they want it, as it’s not our problem.”

"Interview 1"

“If someone who is 12 or so comes, I don’t give them snus. They should at least be in ninth grade and be snus users… I don’t think I would give them something that would make them addicted.”

"Interview 3"

Girls reported that it seldom occurred that someone asked them for snus, so they rarely had to treat others to it, and they did not feel they influenced others to start.

The views of the adolescents were **ambivalent** about whether they should continue with snus or not in the future. They felt it must be their own decision to quit, and nobody else could influence them. But a strong reaction or demand from family or girlfriend could lead to an attempt to quit. Another reason could be to improve their economy. Health reasons were not strong arguments, and as they did not experience many disadvantages from using snus, most of them were not very motivated to quit. The girls were more divided regarding future use of snus, and many of them said that it was difficult for them to know whether or not they would quit in case they got pregnant.

“I think I will actually continue using snus for the rest of my life.”

"Interview 1"

“The idea is that I will be free from nicotine later… by the summer holidays.”

"Interview 5"

## Discussion

The study shows that the process of becoming a snus user contains several steps. As a beginner, you have to endure a number of physical symptoms such as nausea, dizziness and vomiting, which resolve after training some time. Certain circumstances are required for becoming a snus user – friends who use snus, access to snus, and that using snus becomes an important part to the person’s identity. Snus is most often used in a social context that promotes participation and belonging to a group. The picture becomes more complicated by an addiction that develops gradually.

This study shows that being a snus user functions as a social identity and can be seen as an expression of belonging to a group and be like one’s friends. Studies have also shown that adolescents start smoking, and continue smoking, to develop a desired social identity among important groups of friends [22]. The peer group is important for socialisation of the adolescent, while they try to find out what works in different social contexts and for themselves. They adapt their behaviour to that of others in the same category, which is an oblivious process. Many adolescents believe that it is more important to imitate peers than adults [23]. According to Tajfel’s theory on social identity, people identify with those they feel are most like themselves, and join the group that positively affects their social identity [24]. An individual selects his/her social identity based on what is in agreement with his/her expectations and subjective norms. This is also confirmed by our results that the adolescents reported that using snus is part of the picture regarding their choice of profession. Friends give support to and nourish their new identity as a future grown-up [25]. The adolescents in the study also reported that “all” the friends were using snus around them, and that it was their own choice and that their parents had no say in this.

The adolescents were at an age when their identities were developed and they had selected professions that were traditionally male, and the boys felt that using snus was something very manly and closely connected to the future professional role, such as being a farmer or a car mechanic. In Sweden, using snus is considered traditional manly behaviour, which is not the case for smoking, and this is confirmed by studies on tobacco habits [1,3]. The study found that it was important to the boys to identify and position themselves as “a real man”, which in part was demonstrated by the use of snus. According to Connell’s theory on the hierarchy of masculinity, there is an overall culturally and collectively preserved male norm based on a historical ideal on what a “real man” should like and how he should behave [26]. The boys said that girls who used snus were not attractive. This could possibly be interpreted as a male desire to keep the snus as a symbol of masculinity, and that girls should not be associated with “their” symbol. Boys thought that girls who used snus were unwomanly, and it was more accepted if they smoked. This is coherent with a cultural stereotype image in Sweden, and possibly in most countries in the world, about was is considered manly and womanly [27].

With their use of snus, the girls wanted to convey that they were independent and had an identity of their own. It made them different and special, and they described themselves as “tomboys”. The girls expressed their desire to revolt against the norm that it is manly to use snus. This may be a sign of liberation, a diversion from the expected picture of how girls should be. There are rules for how a man and a woman should be, but the social construction is created and re-created depending on the culture we live in [28].

The results demonstrated that the perceived expectations by the adolescents of important people around them made them try snus and eventually learn to like it. To start smoking was not viewed as an alternative. Snus was the first choice as the attitudes from their surrounding were seen as positive. These circumstances are in agreement with Ajzen’s Theory of Planned Behaviour (TPB) [14]. The positive attitude towards using snus, as well as the experience of subjective norms, were the strongest factors that made it easier for them to start using snus. The adolescents’ experience that everybody around them was using snus strengthens the theory that attitudes and norms lead to intentions and behaviour. The adolescents had also considered the consequences of their beliefs, and this influenced their attitudes to the behaviour [14].

The adolescents in the study described how they gradually got stuck in an addiction and were unaware of the fact that the abstinence symptoms they felt could be nicotine addiction. Many of those interviewed had their own experiences of addiction and abstinence symptoms, which they found difficult to endure. A study has shown that early symptoms of addiction are important to the development of tobacco use, and that adolescents find it hard to understand what abstinence symptoms are and what they mean [29]. Adolescents addicted to nicotine do not need to be daily users of tobacco. Just feeling a strong craving for nicotine is reason enough to smoke a cigarette. The first symptom of addiction starts with a strong desire to smoke, followed by nicotine abstinence, which leads to smoking more and more often until you eventually become a daily smoker with an addiction and problems to control the smoking [29]. There is also a strong connection between early symptoms of nicotine addiction and lifelong smoking [30]. It can be assumed that the process is similar for snus users, but studies on this are lacking. A Swedish study of adolescents showed that snus-using adolescents had a four times higher risk of nicotine addiction compared to smoking adolescents [17].

The parents did not react as strongly as the adolescents had expected, and if they had made it clearer that it was not acceptable to use snus, this would probably have made more of them quit using snus. Similar results were seen in a Swedish study on smoking adolescents who wanted the parents to have explicit non-smoking norms, and that compliance was based on good mutual relationships [31].

In this study, both boys and girls reported using snus less if they were distracted by an activity. Furthermore, the girls said they used snus to control their feelings, to reduce their bad temper or if they were sad or angry. A Swedish study on smoking adolescents highlighted the positive effects of nicotine, that it both “increased the well-being” and could “handle negative emotions” [31]. Girls also reported that smoking was a way to handle stress and negative feelings.

### Limitations of the study

A weakness in the results is that the adolescents only represented the practical upper secondary program, and that there were few girls. However, in academic programs, and among girls, only a minor part of nicotine users prefer snus, making it difficult to recruit informants. The findings are not intended to be generally applied, but rather to give in-depth information on the attitudes and opinions of a group of adolescents. It is up to the reader to decide the extent to which the results can be applied to other groups or circumstances.

The results showed that the interviewed adolescents identified themselves as snus users in their future professional roles. For an added dimension in the results, it would have been interesting to include adolescents from the academic program in the study. On the other hand, the results become more specific with adolescents only from the vocational program.

### Methods discussion

Focus group interview is a qualitative research method, which is used for collection of data on attitudes, experiences and opinions of groups [32]. Through the interviews, knowledge was acquired from discussions between adolescents, who were given the opportunity to describe and discuss their snus use habits in their own words. The method gave insight into what it is like to be a snus user and how it started. Since focus group interviews rely on discussions among participants, group members may influence each other as to how they respond to ideas and comments that arise during the discussion [19]. However, it is important to bear in mind that data acquired from a focus group are group data, which reflect the collective ideas shared and talked about by the group. In a focus group, the participants are in a more natural environment than during individual interviews. They are together with their friends and can both influence and be influenced by each other, which is what happens in real life [19]*.*

The interviews were semi-structured and the discussion was based on open questions made up in advance. Thus, the person conducting the interviews may have influenced how the respondents express their experiences. The questions were not asked in a certain order or literally, which gave room for spontaneity, but still with some structure. To make the group discussion easier, boys and girls were interviewed in separate groups, which is recommended in studies with expected differences between the sexes [19].

The purpose of qualitative content analysis is to acquire both knowledge of and an understanding of the phenomenon studied [21]. As we set out to identify variations with regard to differences and similarities of a text, content analysis with an inductive approach was selected. Graneheim and Lundman highlight the importance of the communication for the interpretation as one of the characteristics of content analysis [21]. Texts based on interviews are formulated through interaction between the respondent and the person conducting the interview. The analysis is an unprejudiced description of the variations by identifying differences and similarities in the text, and they are expressed in categories and themes where context is very essential.

The analysis highlighted characteristic and representative elements in order to increase the dependability of the results. To ensure as high credibility as possible, two of the authors (IE, LL) made the analysis independent of each other.

## Conclusion

This study has several implications for preventive and promotional work. The results showed that development of identity was of major importance when adolescents start using snus. The adolescents were unable to interpret the early symptoms of abstinence problems but subsequently were well aware of being addicted. Once they were stuck in a developed addiction and the creation of an image and identity, it was difficult to stop using snus. These factors are important when considering interventions of normative changes and tobacco prevention in schools as well as among parents. It is important to see snus as an addictive product whose health effects are not researched enough at present. We think that using snus should not be seen as a more healthy alternative to smoking, and parents should be involved in the message of a tobacco-free adolescence. A Totally tobacco-free school time, i.e. that nobody smokes or uses snus in school, contribute to a change of norms and attitudes towards a tobacco-free life, and it furthers a more healthy adolescence.

## Competing interests

IE works with tobacco prevention in the Kronoberg County Council. The authors declare that they have no competing interests relating to this study.

## Authors’ contributions

IE was the main author of the manuscript and involved in all aspects of the study. LL participated in the interviews and analyses. MT, GE, and LL were co-authors and provided scientific oversight and feedback throughout the development of the study and this article. All authors read and approved the final version of the article.

## Pre-publication history

The pre-publication history for this paper can be accessed here:

http://www.biomedcentral.com/1471-2458/12/975/prepub
